# Dataset of pesticide and trace metal concentrations in the pollen provisions of wild bees and surrounding soils across European bee hotels

**DOI:** 10.1016/j.dib.2026.113039

**Published:** 2026-06-27

**Authors:** Antoine Gekière, Floriane Jacquemin, Denis Lebailly, Quentin Meekers, Danaé De Blieck, Maria Guadalupe Diaz, Julien Jeuniaux, Michael Van Cutsem, Bach Kim Nguyen

**Affiliations:** a BeeOdiversity SRL, Avenue Arnaud Fraiteur 15-23, 1050, Brussels, Belgium; b Laboratory of Zoology, Research Institute for Biosciences, University of Mons, Place du parc 20, 700 Mons, Belgium

**Keywords:** Biomonitoring, Cavity-nesting bees, Food store, Heavy metal, Trap nest, Plant protection product, Solitary bees

## Abstract

Wild bee populations are declining worldwide, with pesticides and trace metals identified as major drivers due to their widespread use in agriculture and their multiple anthropogenic sources (e.g., industry, traffic and renewable energy technology). Although studies investigating the effects of these pollutants on wild bees are increasing, most rely on concentrations measured in honeybee matrices to define field-realistic exposure levels. This approach may bias exposure assessments, as honeybees and wild bees differ substantially in their foraging ecology, potentially leading to different patterns of pollutant accumulation in pollen provisions. To address this gap, we deployed 79 bee hotels across four West European countries in various land-use contexts and screened 501 pesticides and seven trace metals, which led to the detection and quantification of 92 distinct pesticides and seven trace metals in pollen provisions collected from wild bees, as well as in soil samples surrounding the bee hotels. Overall, 56 and 68 pesticides were detected in pollen provisions and soil samples, respectively, while the seven screened trace metals were detected in both matrices, although prevalence and concentrations varied across bee hotels and soil samples. This dataset provides empirical field-realistic concentrations of pollutants in wild bee matrices, offering a robust reference for future laboratory and semi-field studies. It improves the ecological relevance and accuracy of exposure assessments in wild bee ecotoxicology and enables the investigation of potential links between soil contamination and pollutant levels in the pollen provisions of wild bees.

Specifications Table

**Table:** 

Subject	Earth & Environmental Sciences
Specific subject area	Pesticide and trace metal residues in the pollen provisions of wild cavity-nesting bees and surrounding soils
Type of data	Table & Graph Raw
Data collection	Bee hotels were deployed across 79 sites across West Europe in February 2025 and pollen provisions were collected from March to July 2025. Pollen samples were pooled by bee hotel across the season. A soil sample was also collected at each site. Pesticide and trace metal residues were sent to Primoris (Ghent, Belgium) and quantified using UPLC-MS/MS and ICP-MS, respectively.
Data source location	Data were collected in 79 sites across West Europe (Belgium: *n* = 39, France: *n* = 35, Poland: *n* = 3, Germany: *n* = 2).
Data accessibility	Repository name: Zenodo Data identification number: 10.5281/zenodo.20844744 Direct URL to data: https://doi.org/10.5281/zenodo.20844744
Related research article	None.

## 1 Value of the Data

• Pesticides and trace metals are major drivers to wild bee population decline. However, exposure assessments still largely rely on concentrations measured in honeybee matrices due to the lack of empirical data from wild bee matrices. By providing concentrations directly measured in the pollen provisions of wild bees, this dataset improves the ecological relevance and accuracy of exposure scenarios in wild bee ecotoxicology.
• This dataset reports concentrations of 56 detected pesticides and seven trace metals detected in the pollen provisions of wild bee species collected from 79 bee hotels across four West European countries. It provides field-realistic exposure data directly measured in wild bee matrices, thereby enabling more accurate and ecologically relevant testing of pollutant effects on wild bees under controlled experimental conditions.
• In addition, this dataset includes concentrations of 68 detected pesticides and seven trace metals measured in soil samples collected around the bee hotels. These data enable the exploration of links between soil contamination and pollutant levels found in the pollen provisions of wild bees.
• This dataset is relevant for (i) ecotoxicological studies assessing the effects of pollutants on wild bees, and (ii) environmental studies investigating contamination levels in both soil and biological matrices. This dataset supports a broad range of research on pollutant exposure in wild bee populations through their foraging plants.

## 2 Background

Wild bee populations are declining worldwide, and pesticides have been highlighted as one of the main drivers of this decline [1,2]. Additionally, pollution by trace metals, which is expected to increase with the growing demand for renewable energy technologies [3], has also been identified as a potential threat to wild bee populations [4].

Consequently, an increasing number of studies have investigated the effects of these pollutants on wild bees [5], including during larval development [[6], [7], [8], [9], [10]]. However, due to the limited availability of data on pollutant concentrations in the pollen provisions of wild bees, most laboratory studies rely on concentrations measured in honeybee matrices. This approach biases ecotoxicological studies as honeybees and wild bees differ in their foraging ecology [11]. To date, data on pollutants in the pollen provisions of wild bees remain scarce and are often restricted to specific groups of pollutants, bee species or geographic regions [12,13]. As a result, there is an urgent need for accessible databases documenting pesticide and trace metal concentrations in the pollen provisions of wild bees. Such data will facilitate the design of ecologically relevant laboratory and semi-field experiments on wild bee species.

To address this gap and support the development of field-realistic exposure scenarios, we present a dataset of 92 pesticides and seven trace metals measured in pollen provisions of wild bees collected from 79 bee hotels and in associated soil samples across four West European countries.

## 3 Data Description

The dataset associated with this article reports concentrations of pesticides and trace metals measured in pollen provisions collected by wild cavity-nesting bees from bee hotels, as well as in associated samples of surrounding soil [14]. The dataset is provided in .xlsx format and is organised into multiple spreadsheets. The “concentration” spreadsheet includes information for each sample, including site ID, country, geographic coordinates (latitude and longitude rounded to the nearest 0.5° to ensure site anonymity), the compound nature (pesticide or metal), the sampled matrix (pollen or soil), the compound name, the compound class (e.g., herbicide), the chemical family (e.g., carboxamide), the measured concentration (ppm) and the associated limits of quantification (LOQ, ppm) and detection (LOD, ppm). The “pollen_sampling” spreadsheet indicates the month during which pollen was collected at each site. The “soil_sampling” spreadsheet provides the sampling date for soil as well as the distance between the sampling location and the bee hotel. The “screened_pesticides” spreadsheet lists the 501 pesticide compounds analysed using UPLC-MS/MS. The “screened_metals” spreadsheet details the seven trace metals quantified using ICP-MS. Finally, the “README” spreadsheet summarizes the structure and content of the dataset to facilitate its reuse.

**Fig. 1:**
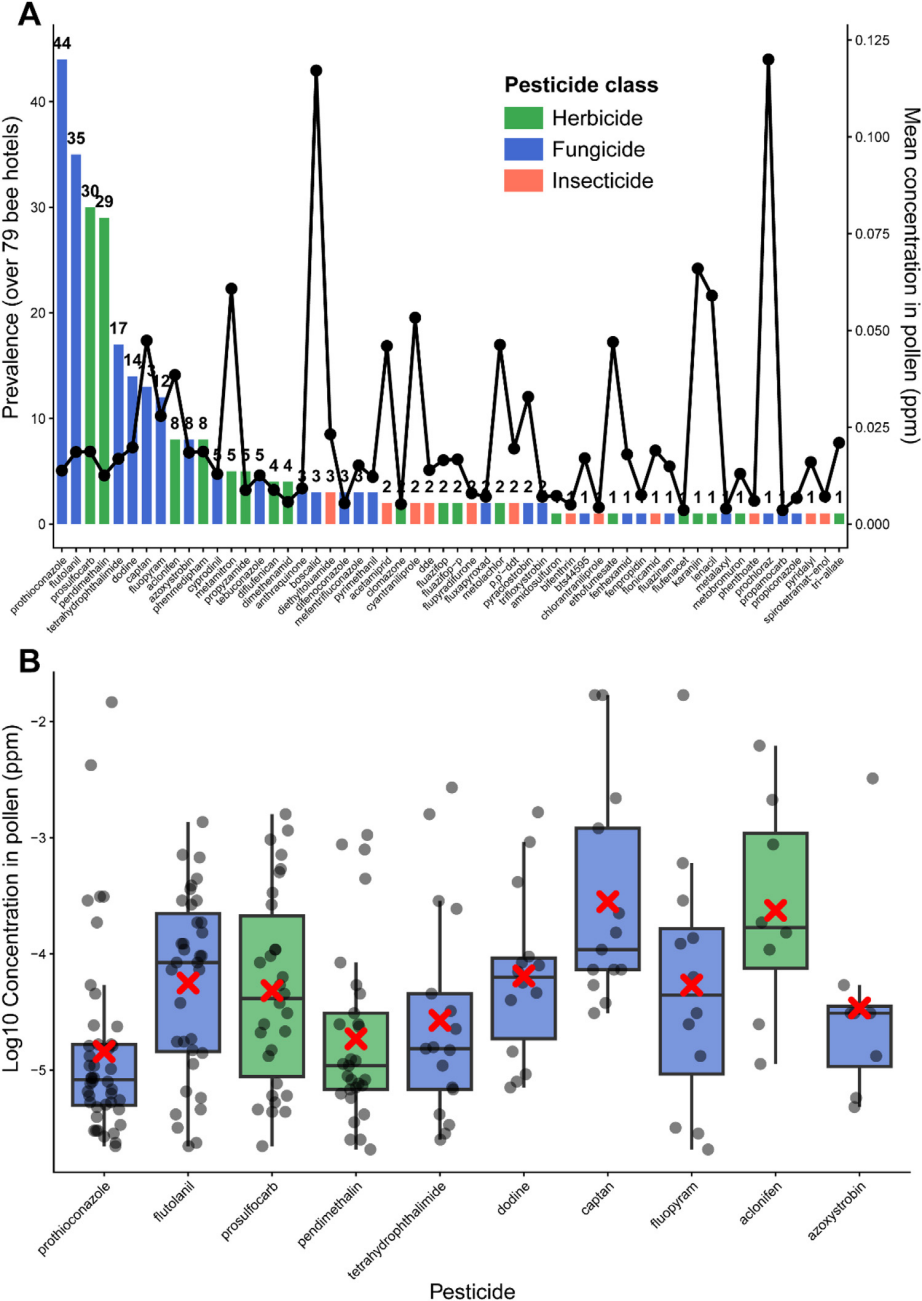
Pesticides detected in pollen provisions collected from 79 bee hotels. **A.** Prevalence and mean concentration (ppm) of each pesticide, with means calculated only from positive (i.e., non-zero concentrations). **B.** Distributions of concentrations (log scale) of the 10 most prevalent pesticides, considering only positive samples (i.e., non-zero concentrations). Crosses indicate the mean concentrations. Colour legend is in panel A.

Among the 79 pollen samples, 56 of the 501 screened pesticides were detected. Pesticide prevalence ranged from one to 44 occurrences across samples, and mean concentrations in positive samples ranged from 0.0036 to 0.1200 ppm (Fig. 1A). The ten most prevalent pesticides detected in pollen were prothioconazole (*n* = 44, min = 0.0035, max = 0.1600, mean = 0.0138 ppm), flutolanil (*n* = 35, min = 0.0035, max = 0.0570, mean = 0.0186 ppm), prosulfocarb (*n* = 30, min = 0.0035, max = 0.0610, mean = 0.0187 ppm), pendimethalin (*n* = 29, min = 0.0034, max = 0.0510, mean = 0.0126 ppm), tetrahydrophthalimide (*n* = 17, min = 0.0037, max = 0.0767, mean = 0.0169 ppm), dodine (*n* = 14, min = 0.0058, max = 0.0620, mean = 0.0198 ppm), captan (*n* = 13, min = 0.0110, max = 0.1700, mean = 0.0474 ppm), fluopyram (*n* = 12, min = 0.0034, max = 0.1700, mean = 0.0279 ppm), aclonifen (*n* = 8, min = 0.0071, max = 0.1100, mean = 0.0385 ppm) and phenmedipham (*n* = 8, min = 0.0046, max = 0.0820, mean = 0.0187 ppm; Fig. 1B).

**Fig. 2:**
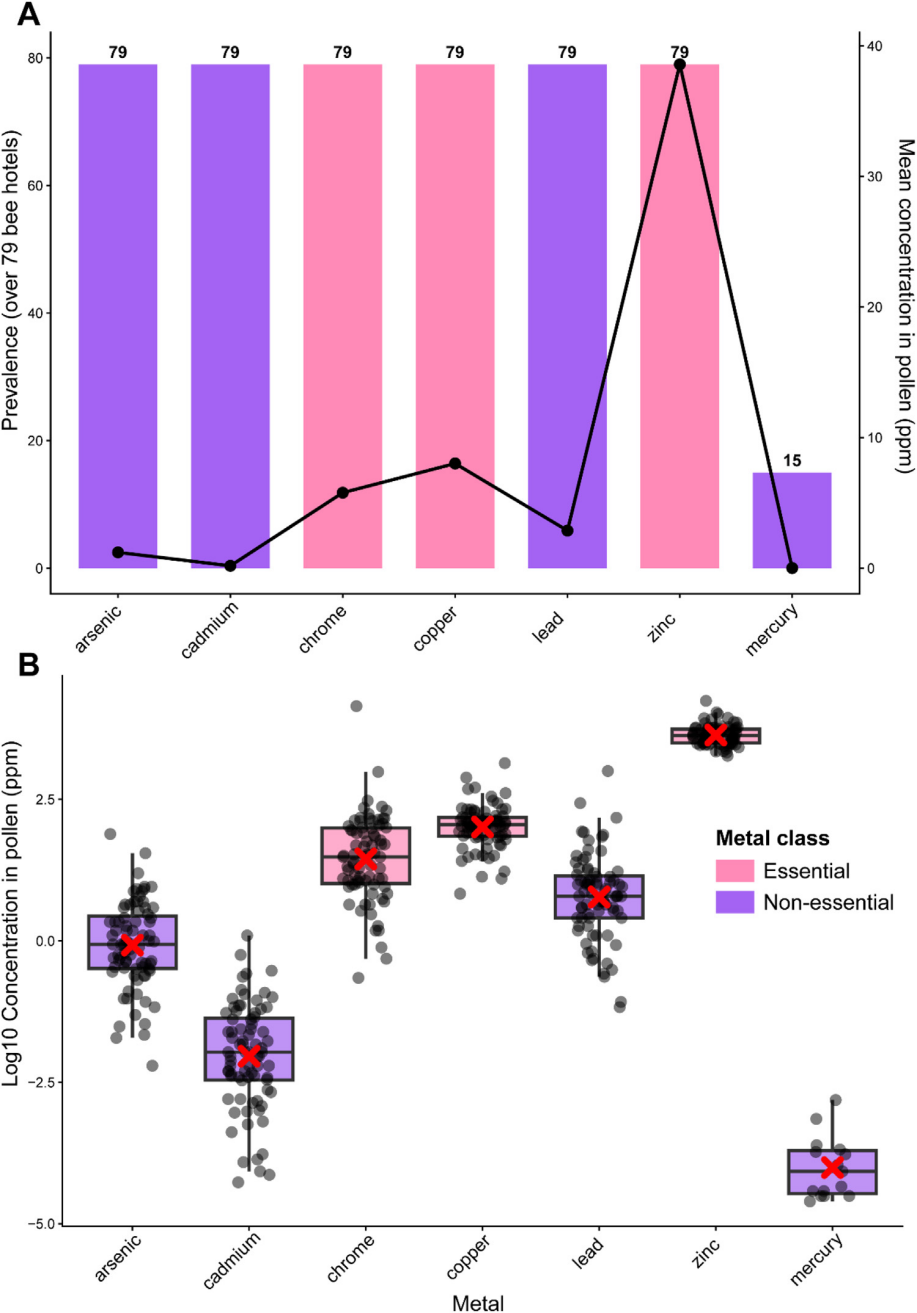
Trace metals detected in pollen provisions collected from 79 bee hotels. **A.** Prevalence and mean concentration (ppm) of each metal, with means calculated only from positive samples (i.e., non-zero concentrations). **B.** Distributions of concentrations (log scale) of the trace metals, considering only positive samples (i.e., non-zero concentrations). Crosses indicate the mean concentrations. Colour legend is in panel B.

All seven screened trace metals were detected in at least one of these 79 pollen samples. Trace metal prevalence ranged from 15 to 79 occurrences across samples, and mean concentrations in positive samples ranged from 0.021 to 38.581 ppm (Fig. 2A). The prevalence and concentration of trace metals were as follows: cadmium (*n* = 79, min = 0.014, max = 1.100, mean = 0.190 ppm), chrome (*n* = 79, min = 0.520, max = 63.300, mean = 5.789 ppm), copper (*n* = 79, min = 2.300, max = 23.100, mean = 8.024 ppm), zinc (*n* = 79, min = 26.300, max = 69.500, mean = 38.581 ppm), arsenic (*n* = 79, min = 0.011, max = 6.600, mean = 1.220 ppm), lead (*n* = 79, min = 0.310, max = 20.100, mean = 2.886 ppm) and mercury (*n* = 15, min = 0.010, max = 0.060, mean = 0.021 ppm; Fig. 2B).

**Fig. 3:**
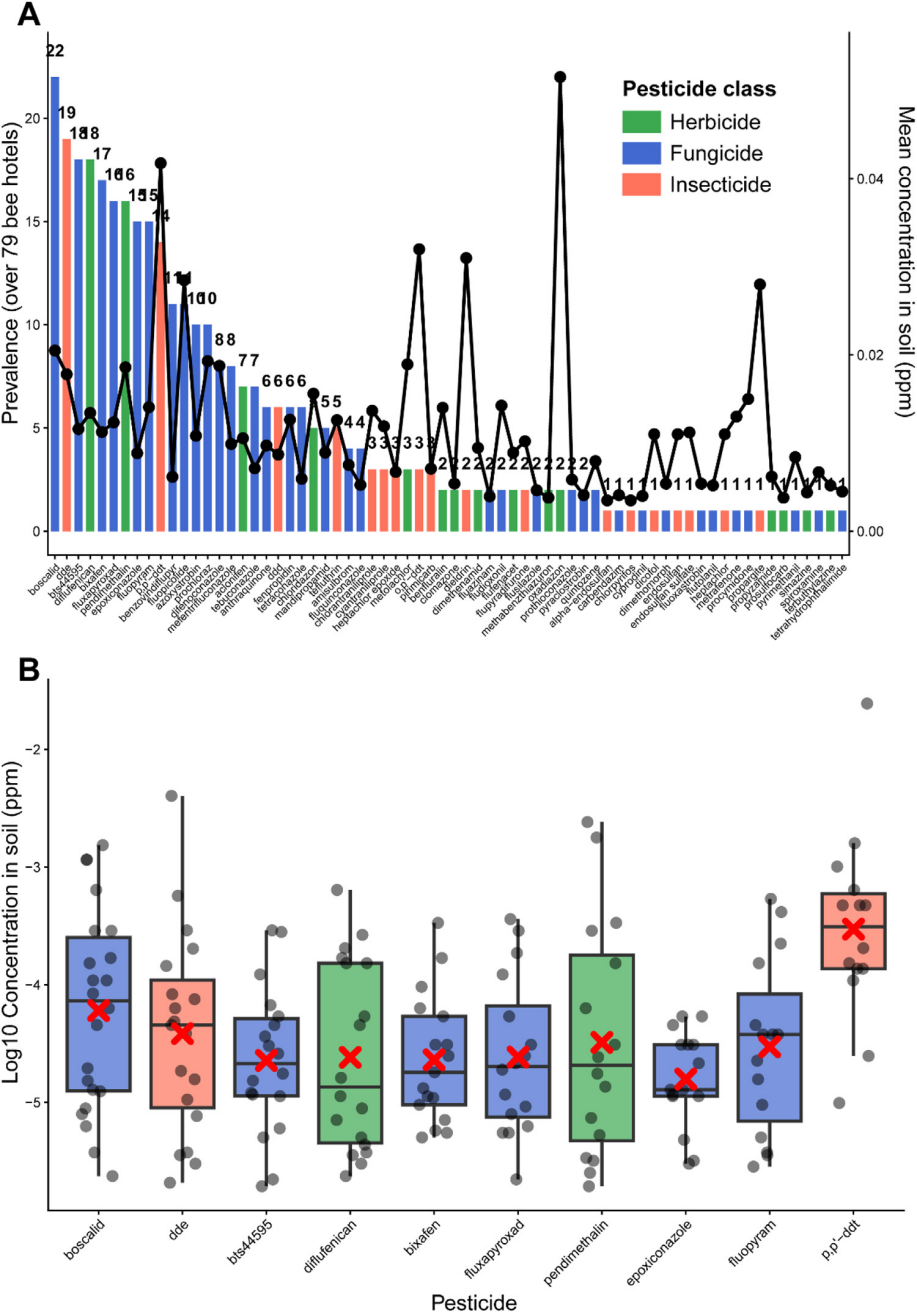
Pesticides detected in soil collected from 79 soil samples. **A.** Prevalence and mean concentration (ppm) of each pesticide, with means calculated only from positive samples (i.e., non-zero concentrations). **B.** Distributions of concentrations (log scale) of the 10 most prevalent pesticides, considering only positive samples (i.e., non-zero concentrations). Crosses indicate the mean concentrations. Colour legend is in panel A.

Among the 79 soil samples, 68 of the 501 screened pesticides were detected. Pesticide prevalence ranged from one to 22 occurrences across samples, and mean concentrations in positive samples ranged from 0.0035 to 0.0516 ppm (Fig. 3A). The ten most prevalent pesticides detected in soil were boscalid (*n* = 22, min = 0.0036, max = 0.0600, mean = 0.0205 ppm), DDE (*n* = 19, min = 0.0034, max = 0.0912, mean = 0.0178 ppm), diflufenican (*n* = 18, min = 0.0036, max = 0.0410, mean = 0.0134 ppm), BTS44595 (*n* = 18, min = 0.0033, max = 0.0291, mean = 0.0116 ppm), bixafen (*n* = 17, min = 0.0050, max = 0.0310, mean = 0.0113 ppm), pendimethalin (*n* = 16, min = 0.0033, max = 0.0730, mean = 0.0186 ppm), fluxapyroxad (*n* = 16, min = 0.0035, max = 0.0320, mean = 0.0123 ppm), fluopyram (*n* = 15, min = 0.0039, max = 0.0380, mean = 0.0141 ppm), epoxiconazole (*n* = 15, min = 0.0040, max = 0.0140, mean = 0.0089 ppm) and P,P'-DDT (*n* = 14, min = 0.0067, max = 0.2000, mean = 0.0418 ppm; Fig. 3B).

**Fig. 4:**
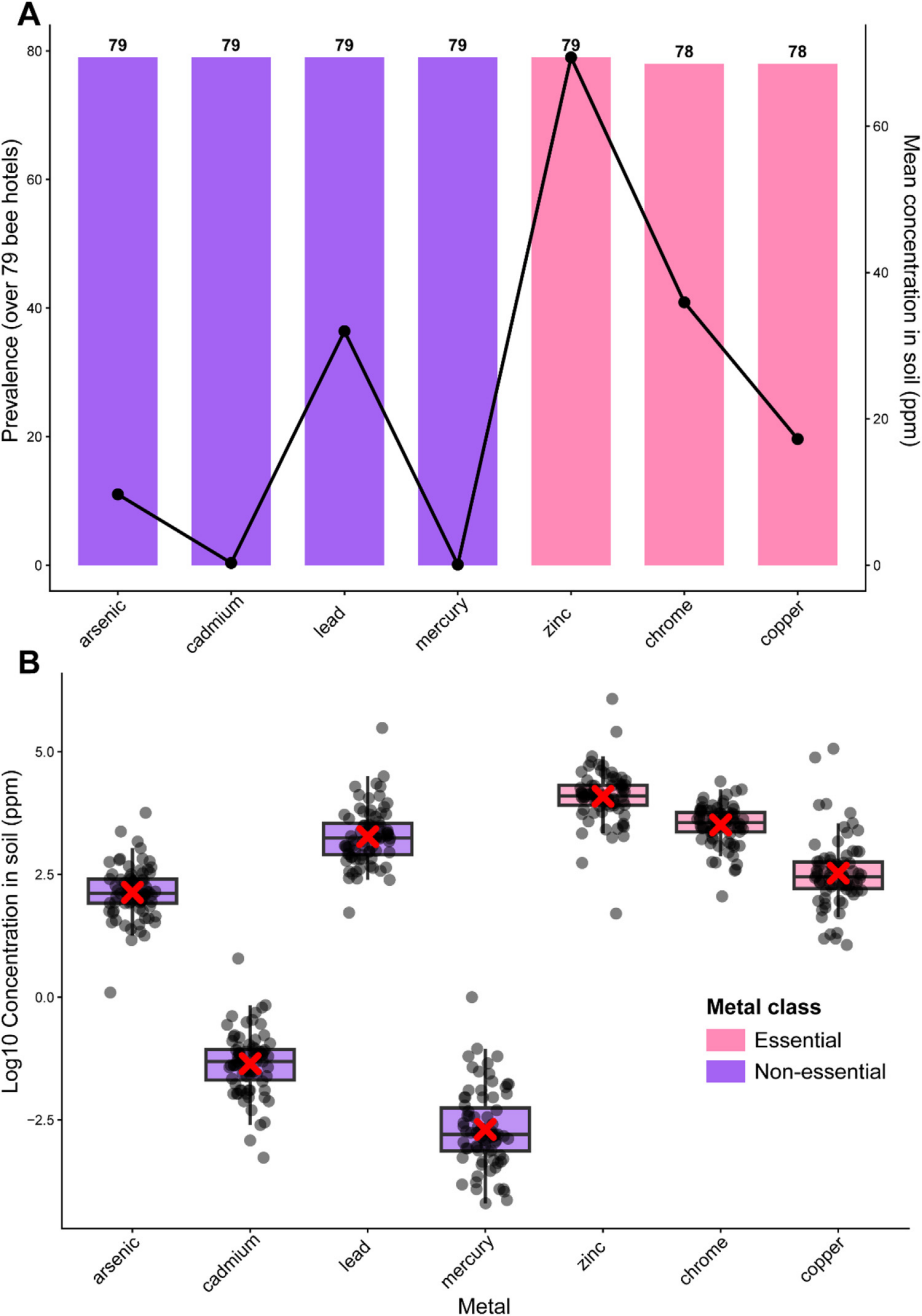
Trace metals detected in soil collected from 79 soil samples. **A.** Prevalence and mean concentration (ppm) of each metal, with means calculated only from positive samples (i.e., non-zero concentrations). **B.** Distributions of concentrations (log scale) of the trace metals, considering only positive samples (i.e., non-zero concentrations). Crosses indicate the mean concentrations. Colour legend is in panel B.

All seven screened trace metals were detected in at least one of these 79 soil samples. Trace metal prevalence ranged from 78 to 79 occurrences across samples, and mean concentrations in positive samples ranged from 0.097 to 69.408 ppm (Fig. 4A). The prevalence and concentration of trace metals were as follows: cadmium (*n* = 79, min = 0.038, max = 2.200, mean = 0.318 ppm), mercury (*n* = 79, min = 0.015, max = 1.000, mean = 0.097 ppm), arsenic (*n* = 79, min = 1.100, max = 42.800, mean = 9.696 ppm), zinc (*n* = 79, min = 5.500, max = 436.000, mean = 69.408 ppm), lead (*n* = 79, min = 5.600, max = 241.000, mean = 31.982 ppm), copper (*n* = 78, min = 2.900, max = 158.000, mean = 17.245 ppm) and chrome (*n* = 78, min = 7.800, max = 81.100, mean = 35.929 ppm; Fig. 4B).

## 4 Experimental Design, Materials and Methods

### 4.1 Sites, bee hotels and sampling

**Fig. 5:**
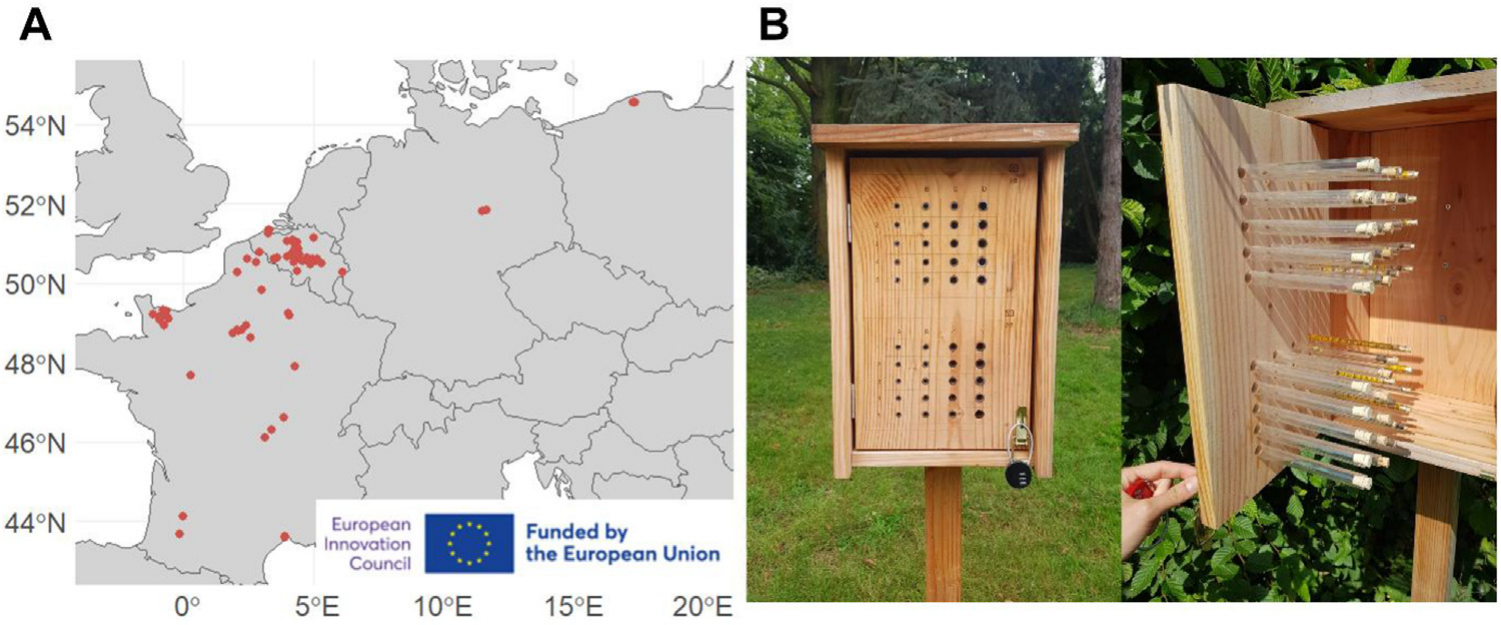
Experimental design. **A.** Spatial distribution of the 79 bee hotels deployed across four Western European countries. **B.** Close-up view of a bee hotel, showing nesting tubes of different diameters, with some tubes filled with pollen collected by cavity-nesting bees.

In February 2025, bee hotels (European patent EP4250914) were installed at 79 sites across Belgium (*n* = 39), France (*n* = 35), Poland (*n* = 3) and Germany (*n* = 2; Fig. 5A). At each site, a single bee hotel was installed at a height of 1.5 m, oriented towards the southeast in an open unshaded area. Each bee hotel consisted of a rectangular wooden box (40 × 30 × 21 cm) containing 40 holes, distributed evenly across four diameters: 4, 6, 8 and 10 mm (i.e., 10 holes per diameter). Plexiglass tubes of corresponding diameters (15 cm length) were inserted behind each hole to allow nest construction by cavity-nesting bees (Fig. 5B). Bee hotels were monitored from March to July 2025. Each month, occupied tubes were removed, replaced with empty ones, and transported to the laboratory for further processing. In addition, one soil sample was collected at each site from the top 17 cm of soil within a maximum distance of 355 m from the bee hotel. Soil was excavated using a clean spade, homogenised in a clean bucket and then compacted into a 125 mL container (∼120 g). All tube and soil samples were stored at −20 °C until further analyses.

### 4.2 Pesticide and trace metal analyses

In the laboratory, for each bee hotel, pollen provisions from tubes of all possible sizes and sampling months were extracted using a micro-spoon and pooled into a single composite sample (i.e., one sample per bee hotel). This approach ensured sufficient material for subsequent chemical analyses (approximately 2 g for trace metal analysis and 5 g for pesticide analysis). Pesticide (including some metabolites) and trace metal concentrations in pollen and soil samples were quantified by Primoris (Ghent, Belgium; BELAC accreditation 057-TEST; ISO/IEC 17,025:2017) using multi-residue analytical methods. Pesticides (and metabolites) were analysed using ultra-performance liquid chromatography coupled with tandem mass spectrometry (UPLC-MS/MS) while trace metals were measured using inductively coupled plasma mass spectrometry (ICP-MS). The analytical screening covered 501 pesticide residues (standard list proposed by Primoris, including metabolites as well as currently approved and not approved substances under EU regulations) and seven trace metal elements. The dataset provides a list of all analysed pesticides and metals, together with the limit of detection (LOD) and limit of quantification (LOQ) for each compound. All the values reported in this manuscript are above LOD, but some reported values are below LOQ.

### 4.3 Graphics

All graphical representations were conducted in the open-source software R version 4.4.0 [15]. The map was plotted using the packages *sf* [16] and *rnaturalearth* [17], while barplots and boxplots were plotted using the packages *ggplot2* [18] and *ggpubr* [19].

## Limitations

The dataset has three main limitations. First, pollen samples were pooled across multiple tubes within each bee hotel to obtain sufficient material for pesticide and trace metal analyses. As a result, samples likely contained pollen provisions collected by several bee species, each characterised by specific nutritional preferences and foraging ranges. Furthermore, because the dataset is based exclusively on cavity-nesting bees, it may not accurately reflect exposure experienced by ground-nesting bees [20]. Consequently, the dataset does not allow species-specific assessments of pesticide and trace metal exposure, and any extrapolation of the results should be done with caution. Second, pollen provisions were not collected from each bee hotel every month, as some hotels contained only empty tubes (i.e., no nesting activity occurred during certain periods). Consequently, the measured pesticide and trace metal concentrations in pollen provisions may not fully represent the entire March-July sampling period. However, the dataset specifies months during which samples were collected for each bee hotel, allowing future users to apply appropriate temporal filtering. Third, soil samples were not collected at a standardised distance from bee hotels nor at a standardised date, although all sampling locations were within the typical foraging range of solitary bees (i.e., approximately 350 m) [21]. As a result, the pesticide and trace metal concentrations in soil may not fully reflect the broader environmental conditions surrounding each bee hotel and may be influenced by the sampling period. The dataset includes information on the distance between soil sampling locations and the corresponding bee hotel as well as the sampling date, enabling users to apply appropriate filtering.

## Ethics Statement

The authors have read and followed the ethical requirements for *Data in Brief*. This work contains no data on human subjects, animals or data collected from social media platforms.

## CRediT Author Statement

**AG:** Conceptualization, Validation, Formal Analysis, Data Curation, Visualisation, Writing - Original Draft, Writing - Review & Editing. **FJ:** Conceptualization, Methodology, Supervision, Writing - Review & Editing. **DL:** Conceptualization, Software, Writing - Review & Editing. **QM:** Conceptualization, Supervision, Resources, Writing - Review & Editing. **DDB:** Conceptualization, Methodology, Writing - Review & Editing. **MGD:** Conceptualization, Methodology, Investigation, Resources, Writing - Review & Editing. **JJ:** Conceptualization, Methodology, Investigation, Resources, Writing - Review & Editing. **MVC:** Conceptualization, Resources, Project administration, Funding acquisition, Writing - Review & Editing. **BKN:** Conceptualization, Resources, Project administration, Funding acquisition, Writing - Review & Editing.

## Data Availability

ZenodoBeeOdiversity dataset - Pesticide and trace metal concentrations in the pollen provisions of wild bees and surrounding soils (Original data)
